# Bis(4-ethoxy­anilinium) sulfate trihydrate

**DOI:** 10.1107/S1600536809036290

**Published:** 2009-09-26

**Authors:** Xue-qun Fu

**Affiliations:** a Ordered Matter Science Research Center, Southeast University, Nanjing 210096, People’s Republic of China

## Abstract

The structure of the title compound, 2C_8_H_12_NO^+^·SO_4_^2−^·3H_2_O, consists of organic layers, water mol­ecules and SO_4_^2−^ anions which lie within the organic layers. In the crystal, inter­molecular N—H⋯O, N—H⋯S O—H⋯O and O—H⋯S hydrogen bonds, some of which are bifurcated, stabilize the structure.

## Related literature

For background to this study, see: Hang *et al.* (2009 ▶); Li *et al.* (2008 ▶). 
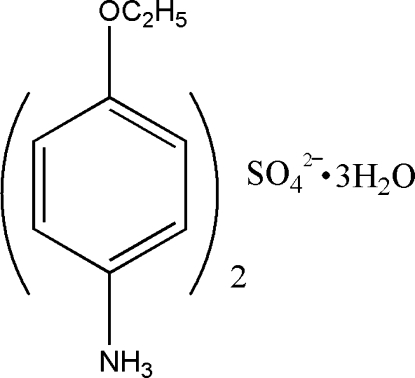


## Experimental

### 

#### Crystal data

2C_8_H_12_NO^+^·SO_4_^2−^·3H_2_O
*M* *_r_* = 426.48
Triclinic, 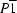

*a* = 7.0455 (14) Å
*b* = 10.969 (2) Å
*c* = 13.787 (3) Å
α = 101.40 (3)°
β = 94.53 (3)°
γ = 90.18 (3)°
*V* = 1041.0 (4) Å^3^
*Z* = 2
Mo *K*α radiation
μ = 0.21 mm^−1^
*T* = 298 K
0.20 × 0.20 × 0.20 mm

#### Data collection

Rigaku SCXmini diffractometer
Absorption correction: multi-scan (*CrystalClear*; Rigaku, 2005 ▶) *T*_min_ = 0.96, *T*_max_ = 0.96
10835 measured reflections
4748 independent reflections
3947 reflections with *I* > 2σ(*I*)
*R*_int_ = 0.041

#### Refinement

*R*[*F* ^2^ > 2σ(*F* ^2^)] = 0.054
*wR*(*F* ^2^) = 0.143
*S* = 1.10
4748 reflections
277 parameters
H atoms treated by a mixture of independent and constrained refinement
Δρ_max_ = 0.30 e Å^−3^
Δρ_min_ = −0.62 e Å^−3^

### 

Data collection: *CrystalClear* (Rigaku, 2005 ▶); cell refinement: *CrystalClear*; data reduction: *CrystalClear*; program(s) used to solve structure: *SHELXS97* (Sheldrick, 2008 ▶); program(s) used to refine structure: *SHELXL97* (Sheldrick, 2008 ▶); molecular graphics: *SHELXTL* (Sheldrick, 2008 ▶); software used to prepare material for publication: *PRPKAPPA* (Ferguson, 1999 ▶).

## Supplementary Material

Crystal structure: contains datablocks I, global. DOI: 10.1107/S1600536809036290/jh2104sup1.cif

Structure factors: contains datablocks I. DOI: 10.1107/S1600536809036290/jh2104Isup2.hkl

Additional supplementary materials:  crystallographic information; 3D view; checkCIF report

## Figures and Tables

**Table 1:** Hydrogen-bond geometry (Å, °)

*D*—H⋯*A*	*D*—H	H⋯*A*	*D*⋯*A*	*D*—H⋯*A*
N2—H2*C*⋯O1^i^	0.89	2.05	2.870 (2)	153
N2—H2*C*⋯S1^i^	0.89	2.77	3.649 (2)	172
N2—H2*D*⋯O2^ii^	0.89	2.07	2.788 (2)	137
N2—H2*E*⋯O7*W*^iii^	0.89	1.94	2.819 (3)	169
N1—H1*D*⋯O8*W*^iii^	0.89	2.14	2.823 (2)	133
N1—H1*D*⋯O9*W*^ii^	0.89	2.46	3.166 (3)	136
N1—H1*E*⋯O3^ii^	0.89	1.93	2.785 (2)	162
N1—H1*F*⋯O1^iv^	0.89	2.03	2.849 (2)	152
O7*W*—H7*D*⋯O4^v^	0.81 (4)	2.11 (4)	2.893 (3)	163 (3)
O8*W*—H8*D*⋯O4^vi^	0.75 (3)	2.12 (3)	2.864 (3)	172 (3)
O9*W*—H9*E*⋯O1^vii^	0.92 (4)	2.07 (4)	2.991 (3)	175 (4)
O9*W*—H9*E*⋯S1^vii^	0.92 (4)	2.98 (4)	3.791 (2)	147 (3)
O7*W*—H7*C*⋯O2	0.83 (4)	2.05 (4)	2.851 (3)	164 (3)
O8*W*—H8*C*⋯O3	0.90 (4)	1.94 (4)	2.815 (3)	164 (3)
O8*W*—H8*C*⋯S1	0.90 (4)	3.02 (4)	3.852 (2)	154 (3)
O9*W*—H9*D*⋯O4	1.03 (5)	2.00 (5)	2.981 (3)	158 (4)
O9*W*—H9*D*⋯S1	1.03 (5)	2.81 (5)	3.547 (2)	129 (3)

Symmetry codes: (i) 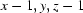; (ii) 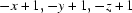; (iii) 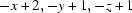; (iv) 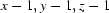; (v) 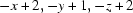; (vi) 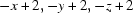; (vii) 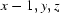.

**Fig. 1.**
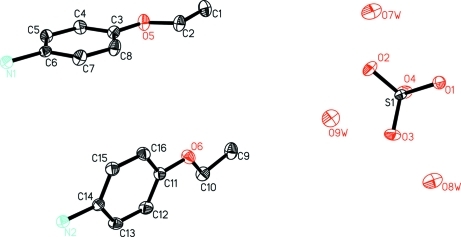
The molecular structure of the title compound, with the atomic numbering scheme. Displacement ellipsoids are drawn at the 30% probability level, and all H atoms have been omitted for clarity.

**Fig. 2.**
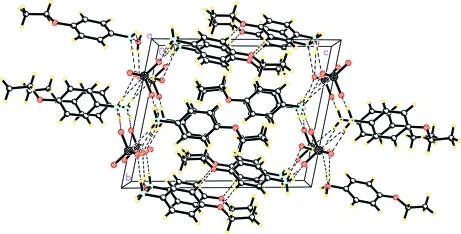
A view of the packing of the title compound, stacking along the a axis. Dashed lines indicate hydrogen bonds.

## supplementary crystallographic information

### Comment

This study is a part of systematic investigation of dielectric-ferroelectric materials, including organic ligands (Li *et al.*, 2008), metal–organic coordination compounds (Hang *et al.*, 2009) and organic–inorganic hybrid. 4-Ethoxyanilinium perchlorate has no dielectric disuniform from 80 K to 480 K, (m.p. 492–493 K).

The asymmetric unit of the title compound contains two 4-ethoxyanilinium cations, one sulfate radical anion and three water molecules (Fig 1). In the anion, the torsion angles of C1—C2—O5—C3 and C9—C10—O6—C11 are -174.8 (2)° and 179.48 (19)°, respectively. The supramolecular structure consists of infinite chains of anions with one cation and three water molecules linked to each anion *via* N—H···O and O—H···O hydrogen bonds.

### Experimental

Single crystals of 4-ethoxyanilinium sulfate are prepared by slow evaporation for five days at room temperature of an ethanol solution of 4-ethoxybenzenamine and sulfuric acid (5 mol l^-^).

### Refinement

Positional parameters of all the H atoms were calculated geometrically and were allowed to ride on the C atoms to which they are bonded, with *U*_iso_(H) = 1.2*U*_eq_(C).

### Crystal data

**Table:**

2C_8_H_12_NO^+^·SO_4_^2^^−^·3H_2_O	*Z* = 2
*M**_r_* = 426.48	*F*(000) = 456
Triclinic, *P*1	*D*_x_ = 1.361 Mg m^−^^3^
Hall symbol: -P 1	Mo *K*α radiation, λ = 0.71073 Å
*a* = 7.0455 (14) Å	Cell parameters from 5008 reflections
*b* = 10.969 (2) Å	θ = 3.0–27.6°
*c* = 13.787 (3) Å	µ = 0.21 mm^−^^1^
α = 101.40 (3)°	*T* = 298 K
β = 94.53 (3)°	Prism, colourless
γ = 90.18 (3)°	0.20 × 0.20 × 0.20 mm
*V* = 1041.0 (4) Å^3^	

### Data collection

**Table:**

Rigaku SCXmini diffractometer	4748 independent reflections
Radiation source: fine-focus sealed tube	3947 reflections with *I* > 2σ(*I*)
graphite	*R*_int_ = 0.041
Detector resolution: 13.6612 pixels mm^-1^	θ_max_ = 27.5°, θ_min_ = 3.0°
ω scans	*h* = −9→9
Absorption correction: multi-scan (*CrystalClear*; Rigaku, 2005)	*k* = −14→14
*T*_min_ = 0.96, *T*_max_ = 0.96	*l* = −17→17
10835 measured reflections	

### Refinement

**Table:**

Refinement on *F*^2^	Primary atom site location: structure-invariant direct methods
Least-squares matrix: full	Secondary atom site location: difference Fourier map
*R*[*F*^2^ > 2σ(*F*^2^)] = 0.054	Hydrogen site location: inferred from neighbouring sites
*wR*(*F*^2^) = 0.143	H atoms treated by a mixture of independent and constrained refinement
*S* = 1.10	*w* = 1/[σ^2^(*F*_o_^2^) + (0.0626*P*)^2^ + 0.4014*P*] where *P* = (*F*_o_^2^ + 2*F*_c_^2^)/3
4748 reflections	(Δ/σ)_max_ = 0.011
277 parameters	Δρ_max_ = 0.30 e Å^−^^3^
0 restraints	Δρ_min_ = −0.62 e Å^−^^3^

### Special details

**Table:**

Geometry. All e.s.d.'s (except the e.s.d. in the dihedral angle between two l.s. planes) are estimated using the full covariance matrix. The cell e.s.d.'s are taken into account individually in the estimation of e.s.d.'s in distances, angles and torsion angles; correlations between e.s.d.'s in cell parameters are only used when they are defined by crystal symmetry. An approximate (isotropic) treatment of cell e.s.d.'s is used for estimating e.s.d.'s involving l.s. planes.
Refinement. Refinement of *F*^2^ against ALL reflections. The weighted *R*-factor *wR* and goodness of fit *S* are based on *F*^2^, conventional *R*-factors *R* are based on *F*, with *F* set to zero for negative *F*^2^. The threshold expression of *F*^2^ > σ(*F*^2^) is used only for calculating *R*-factors(gt) *etc*. and is not relevant to the choice of reflections for refinement. *R*-factors based on *F*^2^ are statistically about twice as large as those based on *F*, and *R*- factors based on ALL data will be even larger.

### Fractional atomic coordinates and isotropic or equivalent isotropic displacement parameters (Å^2^)

**Table:**

	*x*	*y*	*z*	*U*_iso_*/*U*_eq_	
C10	0.0896 (3)	0.6806 (2)	0.58350 (17)	0.0449 (5)	
H10A	−0.0396	0.6635	0.5538	0.054*	
H10B	0.1251	0.7648	0.5791	0.054*	
C13	0.1606 (3)	0.6525 (2)	0.27781 (16)	0.0376 (5)	
H13A	0.0981	0.7046	0.2406	0.045*	
C14	0.2775 (3)	0.56205 (18)	0.23343 (15)	0.0298 (4)	
C11	0.2290 (3)	0.5885 (2)	0.43338 (15)	0.0338 (4)	
C16	0.3460 (3)	0.4968 (2)	0.38719 (16)	0.0405 (5)	
H16A	0.4083	0.4440	0.4238	0.049*	
C12	0.1355 (3)	0.6664 (2)	0.37864 (17)	0.0394 (5)	
H12A	0.0564	0.7276	0.4089	0.047*	
C15	0.3701 (3)	0.4838 (2)	0.28761 (16)	0.0383 (5)	
H15A	0.4487	0.4224	0.2570	0.046*	
N2	0.2998 (2)	0.54499 (16)	0.12718 (12)	0.0323 (4)	
H2C	0.2325	0.6016	0.1020	0.049*	
H2D	0.2583	0.4692	0.0971	0.049*	
H2E	0.4222	0.5538	0.1177	0.049*	
O6	0.2170 (2)	0.59385 (16)	0.53274 (11)	0.0454 (4)	
C7	0.4295 (3)	0.1249 (2)	0.29676 (16)	0.0383 (5)	
H7A	0.5285	0.1591	0.2695	0.046*	
C8	0.4180 (3)	0.1505 (2)	0.39883 (16)	0.0402 (5)	
H8A	0.5088	0.2020	0.4400	0.048*	
C6	0.2953 (3)	0.04922 (18)	0.23606 (14)	0.0284 (4)	
C3	0.2702 (3)	0.09881 (19)	0.43898 (15)	0.0329 (4)	
C5	0.1461 (3)	−0.0017 (2)	0.27536 (15)	0.0348 (5)	
H5A	0.0550	−0.0525	0.2338	0.042*	
C4	0.1337 (3)	0.0235 (2)	0.37657 (16)	0.0365 (5)	
H4A	0.0333	−0.0101	0.4033	0.044*	
N1	0.3073 (2)	0.02291 (16)	0.12851 (12)	0.0316 (4)	
H1D	0.4093	0.0617	0.1140	0.047*	
H1E	0.2031	0.0497	0.0991	0.047*	
H1F	0.3168	−0.0587	0.1070	0.047*	
O4	0.8618 (2)	0.76777 (14)	1.06802 (12)	0.0419 (4)	
O3	0.9964 (2)	0.84402 (14)	0.93687 (12)	0.0411 (4)	
O1	1.2016 (2)	0.76811 (14)	1.05715 (11)	0.0376 (3)	
C9	0.1020 (4)	0.6671 (3)	0.69069 (18)	0.0547 (7)	
H9A	0.0183	0.7250	0.7268	0.082*	
H9B	0.2304	0.6839	0.7192	0.082*	
H9C	0.0651	0.5839	0.6942	0.082*	
C2	0.3919 (3)	0.1835 (2)	0.60656 (16)	0.0435 (5)	
H2A	0.4021	0.2688	0.5976	0.052*	
H2B	0.5136	0.1442	0.5956	0.052*	
S1	1.01303 (6)	0.75062 (4)	0.99930 (3)	0.02523 (14)	
O2	1.0005 (2)	0.62454 (13)	0.93962 (12)	0.0415 (4)	
O5	0.2452 (2)	0.11746 (16)	0.53811 (11)	0.0425 (4)	
C1	0.3390 (4)	0.1809 (3)	0.70955 (17)	0.0509 (6)	
H1A	0.4344	0.2252	0.7572	0.076*	
H1B	0.3307	0.0962	0.7178	0.076*	
H1C	0.2181	0.2195	0.7195	0.076*	
O7W	1.3057 (3)	0.4580 (2)	0.89486 (15)	0.0507 (5)	
O8W	1.2987 (2)	0.98944 (19)	0.89593 (15)	0.0476 (4)	
O9W	0.5193 (3)	0.7333 (2)	0.92324 (17)	0.0644 (5)	
H7D	1.249 (5)	0.393 (3)	0.893 (2)	0.072 (10)*	
H8D	1.255 (4)	1.052 (3)	0.899 (2)	0.050 (9)*	
H7C	1.227 (5)	0.514 (3)	0.900 (2)	0.067 (10)*	
H9E	0.427 (6)	0.747 (4)	0.968 (3)	0.104 (14)*	
H8C	1.209 (5)	0.932 (3)	0.899 (2)	0.077 (10)*	
H9D	0.614 (7)	0.757 (4)	0.985 (3)	0.132 (17)*	

### Atomic displacement parameters (Å^2^)

**Table:**

	*U*^11^	*U*^22^	*U*^33^	*U*^12^	*U*^13^	*U*^23^
C10	0.0433 (13)	0.0460 (13)	0.0452 (13)	0.0036 (10)	0.0118 (10)	0.0049 (10)
C13	0.0376 (11)	0.0343 (11)	0.0436 (12)	0.0107 (9)	0.0059 (9)	0.0127 (9)
C14	0.0276 (9)	0.0277 (10)	0.0337 (10)	−0.0022 (7)	0.0035 (8)	0.0046 (8)
C11	0.0329 (10)	0.0346 (11)	0.0332 (10)	0.0009 (8)	0.0020 (8)	0.0052 (8)
C16	0.0423 (12)	0.0400 (12)	0.0384 (11)	0.0140 (9)	−0.0019 (9)	0.0074 (9)
C12	0.0380 (11)	0.0363 (11)	0.0446 (12)	0.0119 (9)	0.0102 (9)	0.0065 (9)
C15	0.0364 (11)	0.0360 (11)	0.0406 (11)	0.0127 (9)	0.0018 (9)	0.0033 (9)
N2	0.0330 (9)	0.0299 (9)	0.0346 (9)	0.0019 (7)	0.0048 (7)	0.0068 (7)
O6	0.0510 (10)	0.0508 (10)	0.0346 (8)	0.0146 (8)	0.0059 (7)	0.0073 (7)
C7	0.0361 (11)	0.0389 (12)	0.0402 (11)	−0.0116 (9)	0.0133 (9)	0.0047 (9)
C8	0.0392 (11)	0.0429 (12)	0.0354 (11)	−0.0152 (9)	0.0078 (9)	−0.0019 (9)
C6	0.0299 (9)	0.0252 (9)	0.0310 (10)	0.0029 (7)	0.0069 (7)	0.0059 (7)
C3	0.0324 (10)	0.0327 (10)	0.0335 (10)	−0.0011 (8)	0.0081 (8)	0.0045 (8)
C5	0.0317 (10)	0.0363 (11)	0.0362 (11)	−0.0081 (8)	0.0022 (8)	0.0071 (8)
C4	0.0309 (10)	0.0431 (12)	0.0375 (11)	−0.0099 (9)	0.0080 (8)	0.0107 (9)
N1	0.0322 (9)	0.0316 (9)	0.0319 (9)	−0.0001 (7)	0.0067 (7)	0.0068 (7)
O4	0.0408 (9)	0.0383 (9)	0.0515 (9)	0.0061 (7)	0.0238 (7)	0.0126 (7)
O3	0.0387 (8)	0.0403 (9)	0.0517 (9)	0.0021 (6)	0.0062 (7)	0.0261 (7)
O1	0.0327 (8)	0.0346 (8)	0.0450 (9)	−0.0015 (6)	−0.0045 (6)	0.0098 (6)
C9	0.0543 (15)	0.0663 (17)	0.0406 (13)	−0.0081 (13)	0.0121 (11)	0.0005 (12)
C2	0.0406 (12)	0.0485 (13)	0.0379 (12)	−0.0098 (10)	0.0048 (9)	−0.0006 (10)
S1	0.0243 (2)	0.0203 (2)	0.0323 (3)	0.00085 (16)	0.00592 (18)	0.00655 (17)
O2	0.0384 (8)	0.0265 (8)	0.0552 (10)	−0.0019 (6)	0.0070 (7)	−0.0040 (7)
O5	0.0378 (8)	0.0560 (10)	0.0318 (8)	−0.0121 (7)	0.0067 (6)	0.0029 (7)
C1	0.0497 (14)	0.0651 (17)	0.0361 (12)	−0.0007 (12)	0.0038 (10)	0.0052 (11)
O7W	0.0384 (9)	0.0393 (10)	0.0775 (13)	0.0026 (8)	0.0194 (9)	0.0133 (9)
O8W	0.0383 (9)	0.0386 (10)	0.0700 (12)	0.0061 (8)	0.0199 (8)	0.0146 (9)
O9W	0.0516 (11)	0.0690 (14)	0.0738 (14)	0.0023 (10)	0.0175 (11)	0.0121 (11)

### Geometric parameters (Å, °)

**Table:**

C10—O6	1.430 (3)	C3—C4	1.390 (3)
C10—C9	1.510 (3)	C5—C4	1.378 (3)
C10—H10A	0.9700	C5—H5A	0.9300
C10—H10B	0.9700	C4—H4A	0.9300
C13—C14	1.372 (3)	N1—H1D	0.8900
C13—C12	1.394 (3)	N1—H1E	0.8900
C13—H13A	0.9300	N1—H1F	0.8900
C14—C15	1.378 (3)	O4—S1	1.4693 (15)
C14—N2	1.461 (3)	O3—S1	1.4621 (15)
C11—O6	1.369 (3)	O1—S1	1.4861 (15)
C11—C12	1.383 (3)	C9—H9A	0.9600
C11—C16	1.389 (3)	C9—H9B	0.9600
C16—C15	1.376 (3)	C9—H9C	0.9600
C16—H16A	0.9300	C2—O5	1.434 (3)
C12—H12A	0.9300	C2—C1	1.502 (3)
C15—H15A	0.9300	C2—H2A	0.9700
N2—H2C	0.8900	C2—H2B	0.9700
N2—H2D	0.8900	S1—O2	1.4613 (15)
N2—H2E	0.8900	C1—H1A	0.9600
C7—C6	1.371 (3)	C1—H1B	0.9600
C7—C8	1.389 (3)	C1—H1C	0.9600
C7—H7A	0.9300	O7W—H7D	0.81 (4)
C8—C3	1.386 (3)	O7W—H7C	0.83 (4)
C8—H8A	0.9300	O8W—H8D	0.75 (3)
C6—C5	1.384 (3)	O8W—H8C	0.90 (4)
C6—N1	1.463 (2)	O9W—H9E	0.92 (4)
C3—O5	1.367 (2)	O9W—H9D	1.03 (5)
			
O6—C10—C9	107.7 (2)	C8—C3—C4	119.69 (19)
O6—C10—H10A	110.2	C4—C5—C6	119.50 (19)
C9—C10—H10A	110.2	C4—C5—H5A	120.2
O6—C10—H10B	110.2	C6—C5—H5A	120.2
C9—C10—H10B	110.2	C5—C4—C3	120.39 (19)
H10A—C10—H10B	108.5	C5—C4—H4A	119.8
C14—C13—C12	120.0 (2)	C3—C4—H4A	119.8
C14—C13—H13A	120.0	C6—N1—H1D	109.5
C12—C13—H13A	120.0	C6—N1—H1E	109.5
C13—C14—C15	120.56 (19)	H1D—N1—H1E	109.5
C13—C14—N2	120.11 (18)	C6—N1—H1F	109.5
C15—C14—N2	119.29 (18)	H1D—N1—H1F	109.5
O6—C11—C12	124.93 (19)	H1E—N1—H1F	109.5
O6—C11—C16	115.39 (19)	C10—C9—H9A	109.5
C12—C11—C16	119.7 (2)	C10—C9—H9B	109.5
C15—C16—C11	120.4 (2)	H9A—C9—H9B	109.5
C15—C16—H16A	119.8	C10—C9—H9C	109.5
C11—C16—H16A	119.8	H9A—C9—H9C	109.5
C11—C12—C13	119.60 (19)	H9B—C9—H9C	109.5
C11—C12—H12A	120.2	O5—C2—C1	107.52 (19)
C13—C12—H12A	120.2	O5—C2—H2A	110.2
C16—C15—C14	119.79 (19)	C1—C2—H2A	110.2
C16—C15—H15A	120.1	O5—C2—H2B	110.2
C14—C15—H15A	120.1	C1—C2—H2B	110.2
C14—N2—H2C	109.5	H2A—C2—H2B	108.5
C14—N2—H2D	109.5	O2—S1—O3	111.43 (10)
H2C—N2—H2D	109.5	O2—S1—O4	109.70 (10)
C14—N2—H2E	109.5	O3—S1—O4	109.51 (9)
H2C—N2—H2E	109.5	O2—S1—O1	108.58 (9)
H2D—N2—H2E	109.5	O3—S1—O1	108.27 (9)
C11—O6—C10	118.27 (18)	O4—S1—O1	109.31 (10)
C6—C7—C8	120.18 (19)	C3—O5—C2	118.10 (16)
C6—C7—H7A	119.9	C2—C1—H1A	109.5
C8—C7—H7A	119.9	C2—C1—H1B	109.5
C3—C8—C7	119.60 (19)	H1A—C1—H1B	109.5
C3—C8—H8A	120.2	C2—C1—H1C	109.5
C7—C8—H8A	120.2	H1A—C1—H1C	109.5
C7—C6—C5	120.63 (19)	H1B—C1—H1C	109.5
C7—C6—N1	120.03 (17)	H7D—O7W—H7C	108 (3)
C5—C6—N1	119.33 (18)	H8D—O8W—H8C	111 (3)
O5—C3—C8	124.56 (19)	H9E—O9W—H9D	85 (3)
O5—C3—C4	115.74 (18)		
			
C12—C13—C14—C15	0.4 (3)	C6—C7—C8—C3	0.2 (4)
C12—C13—C14—N2	178.24 (19)	C8—C7—C6—C5	0.5 (3)
O6—C11—C16—C15	−179.3 (2)	C8—C7—C6—N1	179.7 (2)
C12—C11—C16—C15	0.4 (3)	C7—C8—C3—O5	179.9 (2)
O6—C11—C12—C13	179.3 (2)	C7—C8—C3—C4	−1.0 (4)
C16—C11—C12—C13	−0.4 (3)	C7—C6—C5—C4	−0.4 (3)
C14—C13—C12—C11	0.0 (3)	N1—C6—C5—C4	−179.62 (19)
C11—C16—C15—C14	−0.1 (3)	C6—C5—C4—C3	−0.4 (3)
C13—C14—C15—C16	−0.3 (3)	O5—C3—C4—C5	−179.7 (2)
N2—C14—C15—C16	−178.2 (2)	C8—C3—C4—C5	1.1 (3)
C12—C11—O6—C10	4.3 (3)	C8—C3—O5—C2	−7.2 (3)
C16—C11—O6—C10	−176.0 (2)	C4—C3—O5—C2	173.6 (2)
C9—C10—O6—C11	179.48 (19)	C1—C2—O5—C3	−174.8 (2)

### Hydrogen-bond geometry (Å, °)

**Table:**

*D*—H···*A*	*D*—H	H···*A*	*D*···*A*	*D*—H···*A*
N2—H2C···O1^i^	0.89	2.05	2.870 (2)	153
N2—H2C···S1^i^	0.89	2.77	3.649 (2)	172
N2—H2D···O2^ii^	0.89	2.07	2.788 (2)	137
N2—H2E···O7W^iii^	0.89	1.94	2.819 (3)	169
N1—H1D···O8W^iii^	0.89	2.14	2.823 (2)	133
N1—H1D···O9W^ii^	0.89	2.46	3.166 (3)	136
N1—H1E···O3^ii^	0.89	1.93	2.785 (2)	162
N1—H1F···O1^iv^	0.89	2.03	2.849 (2)	152
O7W—H7D···O4^v^	0.81 (4)	2.11 (4)	2.893 (3)	163 (3)
O8W—H8D···O4^vi^	0.75 (3)	2.12 (3)	2.864 (3)	172 (3)
O9W—H9E···O1^vii^	0.92 (4)	2.07 (4)	2.991 (3)	175 (4)
O9W—H9E···S1^vii^	0.92 (4)	2.98 (4)	3.791 (2)	147 (3)
O7W—H7C···O2	0.83 (4)	2.05 (4)	2.851 (3)	164 (3)
O8W—H8C···O3	0.90 (4)	1.94 (4)	2.815 (3)	164 (3)
O8W—H8C···S1	0.90 (4)	3.02 (4)	3.852 (2)	154 (3)
O9W—H9D···O4	1.03 (5)	2.00 (5)	2.981 (3)	158 (4)
O9W—H9D···S1	1.03 (5)	2.81 (5)	3.547 (2)	129 (3)

Symmetry codes: (i) *x*−1, *y*, *z*−1; (ii) −*x*+1, −*y*+1, −*z*+1; (iii) −*x*+2, −*y*+1, −*z*+1; (iv) *x*−1, *y*−1, *z*−1; (v) −*x*+2, −*y*+1, −*z*+2; (vi) −*x*+2, −*y*+2, −*z*+2; (vii) *x*−1, *y*, *z*.
